# Hexagonal Boron Nitride Tunnel Barriers Grown on Graphite by High Temperature Molecular Beam Epitaxy

**DOI:** 10.1038/srep34474

**Published:** 2016-09-29

**Authors:** Yong-Jin Cho, Alex Summerfield, Andrew Davies, Tin S. Cheng, Emily F. Smith, Christopher J. Mellor, Andrei N. Khlobystov, C. Thomas Foxon, Laurence Eaves, Peter H. Beton, Sergei V. Novikov

**Affiliations:** 1 School of Physics and Astronomy, University of Nottingham, Nottingham NG7 2RD, UK; 2 School of Chemistry, University of Nottingham, Nottingham NG7 2RD, UK; 3 Nottingham Nanoscale and Microscale Research Centre, University of Nottingham, Nottingham NG7 2RD, UK

## Abstract

We demonstrate direct epitaxial growth of high-quality hexagonal boron nitride (hBN) layers on graphite using high-temperature plasma-assisted molecular beam epitaxy. Atomic force microscopy reveals mono- and few-layer island growth, while conducting atomic force microscopy shows that the grown hBN has a resistance which increases exponentially with the number of layers, and has electrical properties comparable to exfoliated hBN. X-ray photoelectron spectroscopy, Raman microscopy and spectroscopic ellipsometry measurements on hBN confirm the formation of sp^2^-bonded hBN and a band gap of 5.9 ± 0.1 eV with no chemical intermixing with graphite. We also observe hexagonal moiré patterns with a period of 15 nm, consistent with the alignment of the hBN lattice and the graphite substrate.

Multilayer heterostructures based on graphene and hexagonal boron nitride (hBN) have been intensively investigated in recent years^1^,^2^,^3^,^4^,^5^,^6^,^7^,^8^,^9^,^10^,^11^. The crystalline lattices of graphite, graphene and hBN are all hexagonal and are lattice-matched to within 2%. Their electronic properties also complement each other: hBN is a large band-gap (~6 eV) semiconductor, whereas graphite is semi-metallic and graphene (a single sheet of graphite) is a gapless semimetal in which electrons are massless and move with a speed of ~10^6^ m/s^12^. This complementarity has been exploited in several novel ways. For instance, the carrier mobility of graphene is much higher when it is mounted on an atomically-flat substrate of hBN than on SiO_2_ or other dielectrics, for example sapphire^1^. It has also been demonstrated that electrons undergo resonant tunneling in graphene-hBN tunnel transistors when the crystalline lattices of the graphene electrodes are closely aligned^8^. These transistors exhibit gate-tunable negative differential conductance at room temperature, and at low temperatures reveal the chiral properties of the Dirac-Weyl fermions in graphene^9^. Furthermore, the electronic properties of graphene are highly sensitive to its crystalline orientation relative to that of the hBN substrate on which it is mounted: when the two lattices are oriented to within ~1°, a hexagonal “superlattice” is formed with a period of ~14 nm which leads to a quantized energy spectrum of discrete Bloch bands, and, in the presence of a normal magnetic field^5^, a self-similar energy spectrum, the so-called Hofstadter butterfly^13^. In addition, it has been suggested that the graphene undergoes a commensurate-incommensurate structural transition when the graphene and hBN lattices are closely aligned^5^,^14^.

To date, most studies of graphene-hBN heterostructures have been carried out on devices which are made by stacking graphene layers exfoliated from highly-ordered pyrolytic graphite (HOPG) and hBN layers exfoliated from high-quality hBN crystals^15^; these structures are stabilised by van der Waals forces. To scale up the devices and to realize vertical superlattice structures, epitaxial growth of the heterostructures would be highly beneficial. In particular, the alignment of in-plane orientation is necessary to preserve chiral properties of charge carriers in the tunnelling process between graphene layers separated by hBN potential barriers^9^. While there have been several reports on heterostructures formed by the growth of graphene on hBN^16^,^17^,^18^,^19^, there are very few reports of the growth of hBN on graphene or graphite^20^,^21^,^22^ and the major focus of the work to date has been on the use of graphene-terminated metals as substrates using deposition techniques such as chemical vapour deposition^20^, magnetron sputtering^21^ and molecular beam epitaxy^22^.

In this paper, we demonstrate that a van der Waals graphite/hBN heterostructure can be grown using high-temperature plasma-assisted molecular beam epitaxy (MBE) and that this approach provides a promising route to the formation of stacked layered materials with optical and electronic properties comparable with layers exfoliated from bulk hBN. In particular we explore a regime of growth using a much higher substrate temperature than previously been investigated^22^ and also employ an elemental boron source. In addition the direct growth of hBN on a two-dimensional material (HOPG) offers an alternative to chemical vapour deposition^23^,^24^,^25^,^26^ and atomic layer deposition^27^ of hBN on metal substrates; this approach must typically be complemented by complex protocols for the removal and transfer of the grown films. We show that the growth of few-layer crystals of hBN on HOPG is possible and, furthermore, that for the high substrate temperature which we use, the grown layers act as tunnel barriers with a resistance which depends exponentially on layer thickness, and have an optical bandgap of 5.9 eV as determined by ellipsometry, a value which is comparable with bulk material. This development, combined with recent successes in growing graphene on hBN^16^,^28^,^29^, including growth by MBE^30^, offers the prospect for the growth of multilayer graphene-hBN heterostructures to produce a wide variety of quantum well, superlattice and tunnelling devices for future scalable technologies.

## Experimental

Our hBN samples were grown on HOPG substrates using a commercial GENxplor MBE chamber, the base pressure of which was <10^−10^ Torr. This system is equipped with a high temperature heater for the substrate, a high-temperature solid-source effusion cell for boron and a Veeco radio-frequency plasma source for active nitrogen species. Details of the MBE system are described elsewhere^31^. Before introduction into the MBE growth chamber, commercial HOPG substrates with a misorientation (tilt) angle of 0.8°, supplied by Alfa Aesar, were exfoliated using adhesive tape to obtain a fresh surface for growth and subsequently thermally cleaned at ~200 °C in a flow of Ar:H_2_ (95:5) (0.15 standard litres per minute for 4 hours). The hBN samples were all grown with the boron cell maintained at 1875 °C and with the nitrogen source operated at 550 W with a flow rate of 2 sccm, but with different growth times. A fixed substrate heater power is used, giving a substrate thermocouple temperature of 1440 °C which is estimated to correspond to an actual substrate temperature of ~1200 °C (temperature measurement in this system has been discussed previously^31^). Note that high temperature growth of hBN on sapphire by CVD has recently been reported^32^.

The MBE-grown layers were studied by spectroscopic ellipsometry, X-ray photoelectron spectroscopy (XPS) and Raman spectroscopy. The morphology and microscopic structures of hBN domains were determined using atomic force microscopy (AFM) and the electrical properties were measured by conductive AFM (cAFM). These measurements were all performed under ambient conditions. Further experimental details are included in Supplementary Information (SI).

## Results and Discussion

**Figure 1:**
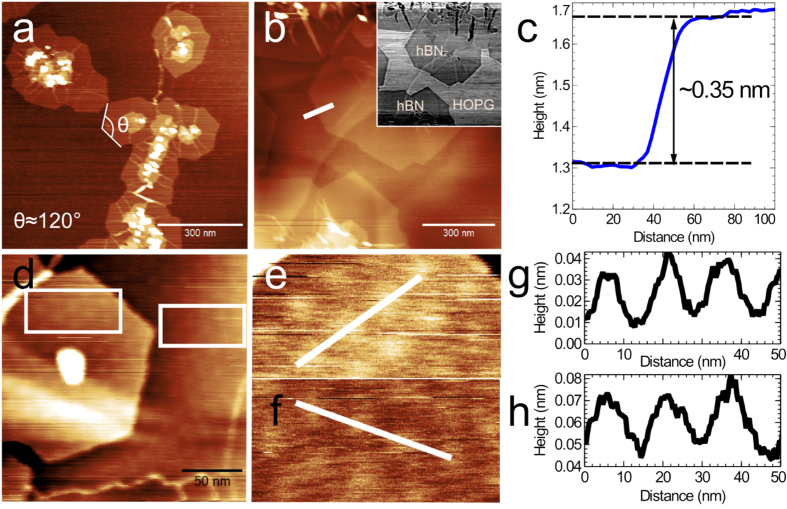
(**a**) AFM image of hBN grown on HOPG for 30 minutes; the facets of each island meet at an angle θ = 120°. (**b**) Surface topographic and (inset) corresponding phase images of hBN grown for 75 minutes on HOPG. (**c**) Line profile for the region indicated in (**b**) showing the step height of monolayer hBN on HOPG. (**d**) AFM image of bilayer hexagonal hBN island on monolayer hBN showing moiré patterns. (**e,f**) Detail of moiré patterns visible in the topography of the regions highlighted by the boxes on the left and right side of d respectively. (**g,h**) Line profiles across the regions highlighted in (**e**,**f**) respectively showing the height variation across the moiré patterns.

AFM images of the surface after growth for 30 and 75 minutes are shown in Fig. 1. In Fig. 1(a) (growth time 30 minutes) we observe the nucleation of islands with typical lateral dimensions of ~100–200 nm which have a hexagonal shape and are highly facetted. The morphology of these islands, with small regions of second and third layers close to their centre, implies that growth is nucleated at these sites. Furthermore, the initial growth is nucleated predominantly at step edges in the HOPG and our AFM images imply that hBN terraces then grow laterally from these points. We also observe bright lines propagating radially in these islands which are possibly due to domain boundaries between regions which are nucleated at different locations and have different rotational order; note that the edges of islands containing such boundaries meet at an angle which deviates from 120^o^, the value expected for hexagonal islands, and observed for many of the growing islands. The overall coverage is estimated to be ~12%.

Figure 1(b) and inset show, respectively, the surface topography and the corresponding phase image (in which contrast arises from difference in material composition), for a sample grown for 75 minutes. For this longer growth time the island size has increased to 200–400 nm and the overall coverage is ~92%. From the topographic images alone it is difficult to distinguish regions of the graphite substrate from the grown material, but this is much clearer in the corresponding phase image in the inset of Fig. 1(b). In the phase image the light/dark contrast regions indicate a different material termination of the surface. The comparison of these images implies that the highly facetted islands have a different composition and can be understood as hBN domains, while the lighter contrast phase regions correspond to the graphite substrate. These regions also correspond to the lowest topographic features in Fig. 1(b). This also allows the identification of a step in the graphite which runs from the lower left of the image; interestingly we find that the grown hBN islands run continuously over this step.

As shown in Fig. 1(c), the step heights of these hBN domains are 0.3–0.4 nm, which is close to the thickness of monolayer (ML) hBN. Thicker hBN layers start to grow on underlying partially-formed hBN monolayers [as shown by the hexagonal bilayer hBN island in Fig. 1(d)], indicating that the hBN growth (at least partly) follows a layer-by-layer mode. Nevertheless, under the growth conditions used to date we do not observe the formation of complete layers before the growth of higher layers as would be expected for the Franck-van der Merwe mode of growth. This may be associated with the nucleation of islands with different in-plane rotational order.

Interestingly, we also observe a moiré pattern in the topographic AFM images of first and second layer hBN as shown in Fig. 1(d–f). The pattern has a period of ~15 nm and a peak to peak amplitude ~30 pm. Moiré patterns have been previously observed in the growth of hBN monolayers on several metal substrates^33^,^34^ and the patterns formed at graphene/hBN interfaces have been discussed by several authors^2^,^5^,^14^. The observed moiré period, ~15 nm, is close to the maximum observed when exfoliated graphene is overlaid on hBN layers; this maximum occurs when the lattices of hBN and graphene are aligned^14^. Our observation is therefore consistent with the formation of an epitaxial hBN layer which is in close alignment with the lattice of the HOPG substrate. The moiré pattern is not observed for all islands; this may be due to the small peak-to-peak amplitude which makes detection difficult in AFM. Alternatively, there may be some rotational disorder in the orientation of the hBN islands.

**Figure 2:**
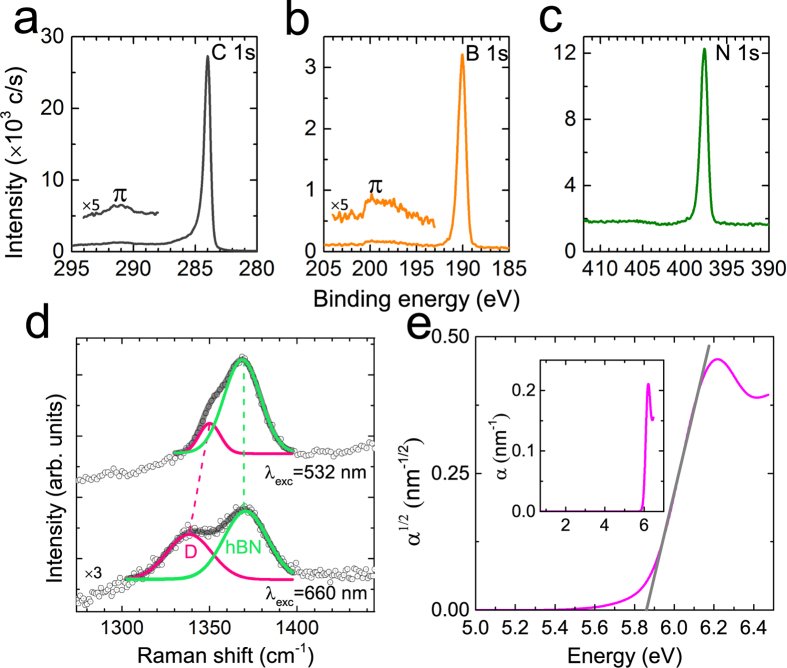
(**a**) C 1s, (**b**) B 1s and (**c**) N 1s XPS spectra of BN grown on HOPG for 3 hours. Note the broad peaks at ~7 eV and ~9 eV from the main peaks in (**a**,**b**), respectively. (**d**) Offset Raman spectra of BN grown on HOPG for 24 hours measured at two different excitation wavelengths. Each spectrum is deconvolved into two Gaussian peaks. Note that the position of the peak assigned to BN (~1370 cm^−1^) is non-dispersive contrary to the *D* peak. (**e**) Optical absorption coefficient, 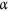, of the BN grown for 3 hours derived from the analysis of spectroscopic ellipsometry measurements. The grey line is used to determine the onset energy of the optical bandgap. Inset of 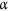 vs. photon energy demonstrates there is no significant absorption in the layer below 5.6 eV.

To investigate the chemical state and bonding of the layers, a sample grown for 3 hours was investigated using XPS (AFM images of this sample are included in SI; the samples were stored under atmospheric conditions over a period of weeks between growth and acquisition of XPS data). The C1s peak due to the HOPG substrate is observed, including a shake-up satellite ~7 eV higher than the main C 1s peak (observed at 284.4 eV) due to 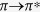 transitions, which indicate the sp^2^-bonding of the HOPG^35^,^36^,^37^. [Fig. 2(a)] The B1s and N1s peaks give the main binding energy positions at 190.3 eV and 397.9 eV, respectively, [Fig. 2(b,c)] which are close to those of hBN^22^,^38^. The expected sp^2^-bonded nature of the hBN is confirmed by the observation of a 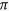-plasmon loss feature at ~9 eV higher binding energy^37^ [Fig. 2(b)]. More importantly, the characteristic shoulder features observed in mixed phases of hBN and C (h-BNC)^39^,^40^ are absent in this sample indicating that the hBN and HOPG are not chemically intermixed. The B/N ratio was estimated to be 0.9 taking into account the integrated peak intensities and sensitivity factors of the elements. This small deviation to apparent N-rich from stoichiometry is likely due to error generated from the use of reference sensitivity factors to calculate the atomic % which are based on a bulk sample of hBN in which the photoelectrons will sample slightly different depths due to their different kinetic energies. In a thin film this will bias the atomic % calculated to increase the apparent amount of the lowest kinetic energy (highest binding energy) elemental peak^41^.

Due to the wide bandgap of hBN, and unlike graphene, the resonance condition of Raman scattering is not met for visible light excitation so that the Raman signal of thin hBN is weak. In order to confirm the hexagonal phase of the hBN by Raman scattering we therefore grew hBN on HOPG for a much longer time (24 hours) so as to increase the probe volume and the Raman intensity. For hBN/graphite heterostructures the *D* peak of graphite and the *E2g* Raman peak of hBN are close in energy. However, the *E2g* mode of hBN is non-dispersive, while the *D* peak of graphite depends on the excitation wavelength^42^. Figure 2(d) shows the Raman spectra of this thicker hBN sample recorded at two different excitation wavelengths (AFM images of this sample are included in SI). The *D* peak located at the lower wave number shifts with the excitation wavelength while no shift is observed for the peak located at ~1370 cm^−1^ [Fig. 1(d)], implying that the sample consists of two chemically separated components, hBN and graphite, which is consistent with the XPS results.

The complex refractive index of the hBN was determined using a variable angle spectroscopic ellipsometer and an incident photon energy ranging from 0.7 to 6.5 eV (see Fig. 2(e) which shows measurements on a sample grown for 3 hours). A commercial software package^43^ was used to model the optical response as the summation of two Gaussian oscillators and a UV pole. The absorption coefficient *α*(*E*) can be calculated as a function of photon energy, *E*, from the optical constants and, as shown in the inset of Fig. 2(e), is characterized by a sharp absorption edge at ~6 eV with no lower energy absorption bands resulting from point defects such as N vacancies and C incorporation^39^,^44^, indicative of a layer with low defect density. Recently, it was reported that hBN is in fact an indirect bandgap semiconductor and the interband optical transition is phonon-assisted^45^. For an indirect bandgap semiconductor, *α*(*E*) can be expressed by *α*(*E*) = *A*(*E−E*_*G*_)^2^, where *A* is a constant and *E*_*G*_ is the optical bandgap^46^. Using a straight line plot of *α*(*E*)^1/2^ versus *E*, we obtain an estimate of the band gap of hBN, *E*_*G*_ = 5.9 ± 0.1 eV. (A good fit is also obtained if the material is modelled as a direct gap semiconductor, *α*(*E*) = *A*(*E−E*_*G*_)^1/2^; with *E*_*G*_ = 6.0 ± 0.1 eV). This value is close to that reported in the literature^45^,^47^. It is also possible to determine the thickness of the hBN layer from ellipsometry measurements; we find 0.94 ± 0.1 nm. These results confirm that the topographic islands observed in Fig. 1 correspond to high quality hBN layers with negligible chemical intermixing with the HOPG substrate. The experimentally-determined value for the bandgap is independent of layer thickness over the range 0.6–2.5 nm which implies that the modification of hBN bandstructure which has recently been reported for hBN grown on nickel^26^ does not occur when using HOPG substrates.

**Figure 3:**
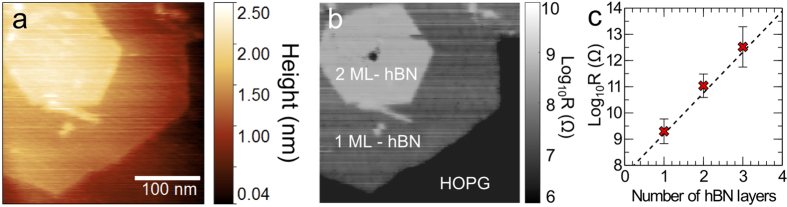
Surface topographic (**a**) and the corresponding conducting AFM (**b**) images of an hBN monolayer and a bilayer grown on HOPG. (**c**) Vertical resistance as a function of the hBN layer thickness determined by performing current-voltage measurements on the hBN surface at different positions on the substrate surface. Note the exponential dependence of the electrical resistance on the number of the hBN layers.

hBN is a large bandgap material and is expected to have a high electrical impedance, a crucial property in its role as a dielectric and as a tunnel barrier in stacked heterostructures of layered two-dimensional materials^48^. To assess the electrical properties of the grown layers we have used cAFM to measure the resistance across the hBN layers using a metal coated cantilever and the HOPG substrate as electrodes. Figure 3(a,b) show spatially correlated maps of the surface topography and the layer resistance for hBN grown on HOPG for 75 minutes. Note that the bright regions in Fig. 3(b) correspond to high resistance and it is clear that the thicker layers are much more resistive. The uniformity of contrast also indicates that the hBN is indeed a layered structure on the conducting HOPG (the more resistive epitaxial layers may be clearly distinguished from the low resistance substrate) and is largely free of defects and is electrically homogeneous.

The current flowing across an hBN tunnel barrier is expected to be exponentially dependent on the barrier thickness^48^,^49^. We have investigated this by measuring the resistance of hBN layers with thicknesses of 1–3 monolayers. The current-voltage I(V) dependence was determined using a conductive AFM cantilever at locations with different thickness as determined from topographic AFM images as shown in Fig. 3(c). The resistance shows the expected exponential dependence on layer thickness. The current decays by a factor ~40 per hBN layer, very close to the value determined for exfoliated hBN layers^48^ confirming the high electrical quality of our grown layers. Note also that the linearity of the plot in Fig. 3(c), combined with agreement with the properties of grown and exfoliated^48^ material further supports the observation above that the electrical properties of the MBE-grown hBN are not strongly dependent on thickness.

## Conclusions

To conclude, we have demonstrated direct epitaxial growth of high-quality hBN layers on graphite substrates using high-temperature plasma-assisted MBE. It was found that the hBN grown on HOPG is hexagonal with no chemical intermixing with the graphite. The resistance of atomically well-resolved hBN layers shows an exponential dependence on the number of the layers and in addition we observe moiré patterns with 6-fold symmetry in our topographic images of the hBN layers, indicating a local alignment of the hBN and graphite lattices. Our results, combined with our recent success in growing graphene on hBN, demonstrate that this is a promising approach for the commensurate growth of multilayer heterostructures based on hBN and graphene. In our future work we plan to explore this growth process in more detail and extend our work to investigate the growth of hBN on graphene, and also to determine the origin of the variation of rotational order and the mechanisms for nucleation. In summary our current results demonstrate that hBN can be grown on a two-dimensional material using MBE and provide an important step towards the epitaxial growth of complex graphene/hBN heterostructures.

## Additional Information

**How to cite this article**: Cho, Y.-J. *et al.* Hexagonal Boron Nitride Tunnel Barriers Grown on Graphite by High Temperature Molecular Beam Epitaxy. *Sci. Rep.* **6**, 34474; doi: 10.1038/srep34474 (2016).

## Supplementary Material

Supplementary Information
