# Corrigendum: Hexagonal Boron Nitride Tunnel Barriers Grown on Graphite by High Temperature Molecular Beam Epitaxy

**DOI:** 10.1038/srep46799

**Published:** 2017-05-08

**Authors:** Yong-Jin Cho, Alex Summerfield, Andrew Davies, Tin S. Cheng, Emily F. Smith, Christopher J. Mellor, Andrei N. Khlobystov, C. Thomas Foxon, Laurence Eaves, Peter H. Beton, Sergei V. Novikov

Scientific Reports 6: Article number: 34474; 10.1038/srep34474 published online: 09 29 2016; updated: 05 08 2017.

As the authors of references 25 and 26 also employed plasma-assisted molecular beam epitaxy for the synthesis of hexagonal boron nitride films, the authors would like to make the following changes to the Introduction section of their Article:

“In addition the direct growth of hBN on a two-dimensional material (HOPG) offers an alternative to chemical vapour deposition^23,24,25,26^ and atomic layer deposition^27^ of hBN on metal substrates; this approach must typically be complemented by complex protocols for the removal and transfer of the grown films”.

Should read:

“In addition the direct growth of hBN on a two-dimensional material (HOPG) offers an alternative to chemical vapour deposition^23,24^, MBE^25,26^ and atomic layer deposition^27^ of hBN on metal substrates; this approach must typically be complemented by complex protocols for the removal and transfer of the grown films”.

