# Supplementary material for: Hexagonal Boron Nitride Tunnel Barriers Grown on Graphite by High Temperature Molecular Beam Epitaxy
Source: Sci Rep. 2016 Sep 29;6:34474. doi: 10.1038/srep34474 (PMC5041098; doi:10.1038/srep34474)
Supplement: Supplementary Information [file srep34474-s1.pdf]

Hexagonal Boron Nitride Tunnel Barriers Grown on Graphite by High Temperature Molecular Beam Epitaxy

Supplementary Information

Yong-Jin Cho,[1] Alex Summerfield,[1] Andrew Davies,[1, 2] Tin S. Cheng,[1]

Emily F. Smith,[2, 3] Christopher J. Mellor,[1] Andrei N. Khlobystov,[2, 3]

C. Thomas Foxon,[1] Laurence Eaves,[1] Peter H. Beton,[1] and Sergei V. Novikov[1]

[1]School of Physics and Astronomy, University of Nottingham, Nottingham NG7 2RD, UK

[2]School of Chemistry, University of Nottingham, Nottingham NG7 2RD, UK

[3]Nottingham Nanoscale and Microscale Research Centre,

University of Nottingham, Nottingham NG7 2RD, UK

X-ray photoelectron spectroscopy (XPS)

For XPS measurements, hBN samples were mounted on standard sample bars with copper clips and analysed with a Kratos AXIS ULTRA with a mono-chromated Al kα X-ray source (1486.6eV) operated at 10 mA emission current and 12 kV anode potential (120 W). A wide (survey) scan (Fig. S1) at pass energy 80 eV and high resolution scans at pass energy 20 eV were acquired on each sample at three positions. The analysis area was approximately 300 × 700 μm^2. The analysis chamber pressure was lower than 8 × 10^{-9} Torr. The peak areas in the wide scan data can be used to calculate the elemental atomic % using Kratos relative sensitivity factors (RSFs). No charge correction was applied. Data processing was carried out using

CASAXPS software (www.casaxps.com) with Kratos sensitivity factors (RSFs) to determine atomic % values from the peak areas.

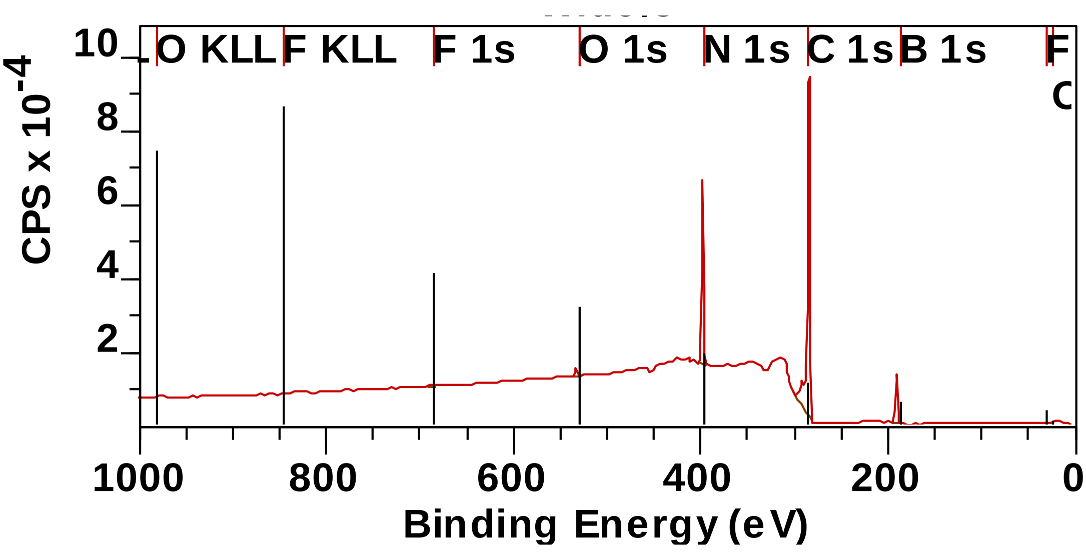

Figure S1. A wide XPS spectrum of hBN grown on HOPG for 3 hours.

Variable angle spectroscopic ellipsometry

The spectroscopic ellipsometric response was measured on a J.A. Woollam M-2000 DI instrument at incident angles of 55˚, 65˚ and 75˚ over the wavelength range of 193 – 1690 nm (712 wavelengths). The HOPG substrate was characterised by measuring the rear of the sample. This ensured the substrate response was from a similar sample of HOPG which had been subjected to the same pretreatment and temperature cycles as the side on which the hBN was grown on. HOPG consists of many grains of graphite which are closely, but not perfectly, oriented. Therefore only 15-20% of the light from the source is specularly reflected into the analyser. However light that is reflected from the sample into the analyser will have hit the sample at the specified angle of incidence.

The optical response of the HOPG substrate shows the expected response of graphite – a Drude-like response in the infra-red, broad absorption in the visible and an exciton peak in the UV. Significantly, there are no sharp features in the optical response around a photon energy of 6 eV.

The variable angle spectroscopic ellipsometric response of the hBN layer on HOPG was modelled in CompleteEase v5.10 (J.A. Woollam Co., Inc.). A good fit was found using two Gaussian oscillators with centre energies of 6.147eV and 6.481 eV and a UV pole at 11eV (default setting). The modelled optical response was then used to calculate the absorption coefficient.

Atomic Force Microscopy (AFM) and Conductive AFM (cAFM)

For AFM imaging, MBE grown hBN on HOPG samples were imaged using an Asylum Research Cypher-S AFM operating in amplitude modulated tapping mode (AC mode) using Multi75Al-G cantilevers (Budget Sensors) (F_0 = 75 +/- 15 kHz, k = ~3 N/m) at amplitude setpoints of 400-600 mV at room temperature.

For cAFM measurements, hBN on HOPG samples were attached to a steel sample holder using conductive silver paint (Ted Pella). Measurements were performed using Pt-Coated Olympus AC240TM conductive cantilevers (Asylum Research) in an Asylum Cypher-S AFM fitted with an ORCA C-AFM cantilever holder (current range +/- 20 nA). Samples were imaged in contact mode at a set-point of 0.2-0.3 V and a bias of 10-100 mV applied to the sample.

For determination of resistance vs hBN layer thickness, samples were first imaged in AC-mode and the thickness of the hBN layers determined using topographic measurements. Point spectra were then taken at regions of different hBN layer thicknesses and the sample bias ramped between +/- 2 V. The resistance was then determined using the linear regions of the I(V) spectra.

AFM Images of Samples used for Ellipsometry, XPS and Raman Measurements

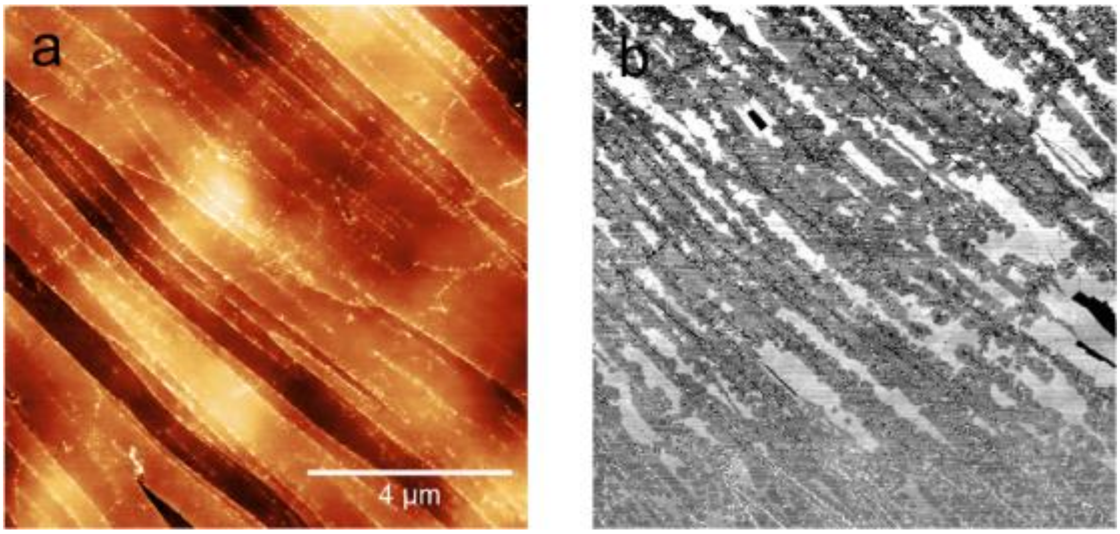

Figure S2. AC-mode AFM image showing a) topography and b) phase-channel of an hBN on HOPG sample grown for 3 h used for XPS and spectroscopic ellipsometry measurements.

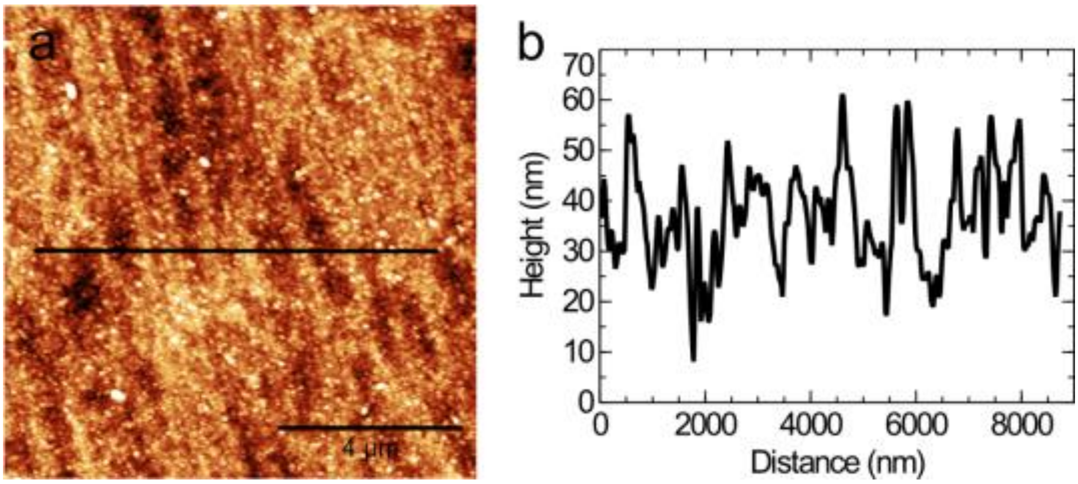

Figure S3. a) AC-mode AFM image of hBN on HOPG sample grown for 24 h and used for Raman spectroscopy measurements. b) Profile across the sample as indicated by the black line in image a.

AFM Images and I(V) measurements for sample used to generate Fig. 3c

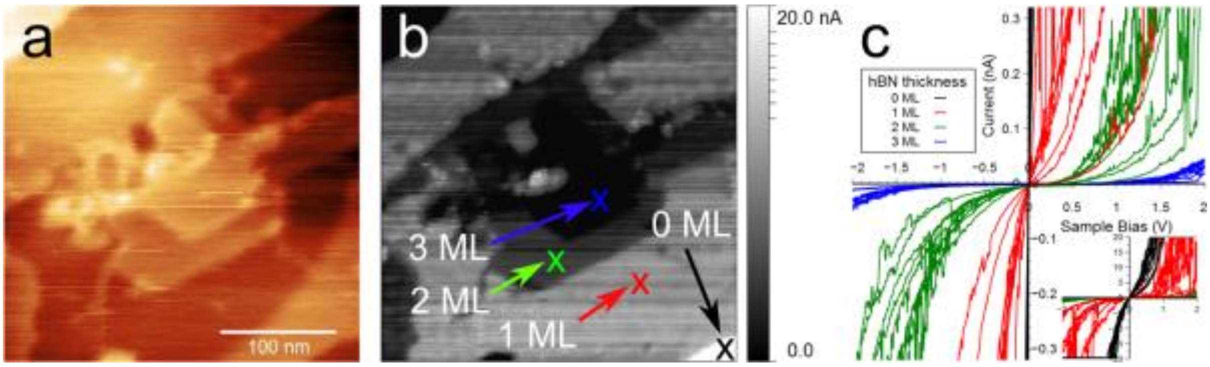

Figure S4 a) topographic AFM image of hBN grown on graphite; b) same are imaged using conducting AFM in which the contrast reflects the resistance of the epitaxial layer. Regions og the graphite substrate (0 ML) and one, two and three monolayers are identified. Also marked are the locations where the I(V) measurements are acquired; these are plotted in c); note the near vertical black line passing through the origin corresponds to the low resistance graphite substrate while the 1, 2 and 3 hBN layers are colour coded red, green and blue respectively. The plot in Figure 3c of the main paper is determined by measuring the slope of the linear region between -0.2 and 0.2V for each trace and then plotting on a log scale leading to the error bars shown in Fig. 3c.
